# Molecular neurobiological markers in the onset of sodium appetite

**DOI:** 10.1038/s41598-022-18220-w

**Published:** 2022-08-20

**Authors:** Cintia Y. Porcari, María J. Cambiasso, André S. Mecawi, Ximena E. Caeiro, José Antunes-Rodrigues, Laura M. Vivas, Andrea Godino

**Affiliations:** 1 Instituto de Investigación Médica Mercedes y Martín Ferreyra (INIMEC-CONICET-Universidad Nacional de Córdoba), Friuli 2434, Barrio Parque Vélez Sarsfield, Casilla de Correo, 389-5000, 5016 Córdoba, Provincia de Córdoba Argentina; 2 Department of Physiology, School of Medicine of Ribeirao Preto, University of Sao Paulo, Sao Paulo, Brazil; 3 Facultad de Ciencias Exactas Físicas y Naturales, Universidad Nacional de Córdoba, Córdoba, Argentina; 4 Facultad de Psicología, Universidad Nacional de Córdoba, Córdoba, Argentina; 5 Laboratory of Molecular Neuroendocrinology, Department of Biophysics, Paulista Medical School, Federal University of São Paulo, São Paulo, Brazil; 6 Departamento de Biología Bucal, Facultad de Odontología, Universidad Nacional de Córdoba, Córdoba, Argentina

**Keywords:** Neuroscience, Endocrinology

## Abstract

Sodium appetite is a motivational state involving homeostatic behavior, seeking the ingest of salty substances after sodium loss. There is a temporal dissociation between sodium depletion (SD) and the appearance of sodium appetite. However, the responsible mechanisms for this delay remain poorly elucidated. In the present study, we measured the temporal changes at two and 24 h after SD in the gene expression of key elements within excitatory, inhibitory, and sensory areas implicated in the signaling pathways involved in the onset of sodium appetite. In SD rats, we observed that the expression of critical components within the brain control circuit of sodium appetite, including Angiotensin-type-1 receptor (Agtr1a), Oxytocin-(OXT-NP)-neurophysin-I, and serotonergic-(5HT)-type-2c receptor (Htr2c) were modulated by SD, regardless of time. However, we observed reduced phosphorylation of mitogen-activated protein kinases (MAPK) at the paraventricular nucleus (PVN) and increased oxytocin receptor (Oxtr) mRNA expression at the anteroventral of the third ventricle area (AV3V), at two hours after SD, when sodium appetite is inapparent. At twenty-four hours after SD, when sodium appetite is released, we observed a reduction in the mRNA expression of the transient receptor potential channel 1gene (Trpv1) and Oxtr in the AV3V and the dorsal raphe nucleus, respectively. The results indicate that SD exerts a coordinated timing effect, promoting the appearance of sodium appetite through changes in MAPK activity and lower Trpv1 channel and Oxtr expression that trigger sodium consumption to reestablish the hydroelectrolytic homeostasis.

## Introduction

The mammalian body requires and maintains a homeostatic extracellular sodium concentration to conduct nerve impulses, contract and relax muscles, and keep the proper water balance, minerals, and blood pressure. It is estimated that about 500 mg of sodium is needed daily for these vital functions^1^. Natremia imbalance and, in particular, hyponatremia is the most frequent electrolyte abnormality observed in hospitalized subjects. It is constantly associated with an increased risk of complications and reduced survival in patients. However, the rapid correction by hypertonic sodium solution infusion even produced more neurological dramatic complications^2^. Thus, knowing the brain components involved in sodium appetite (SA) onset, and the temporal course of their interplay during hyponatremia, becomes highly relevant.

Sodium appetite is a motivational state that involves necessary homeostatic behavior, seeking out and ingesting salty substances to compensate for sodium losses, operationally defined by measuring hypertonic sodium solution consumption under specified experimental conditions^3^. There is a temporal dissociation between sodium depletion (SD) and the appearance of sodium appetite. In contrast to thirst, sodium appetite does not increase until long after the hypovolemia recedes (many hours or days later, according to the experimental model). However, the exact mechanisms responsible for this delay are incompletely understood^4–6^.

**Figure 1:**
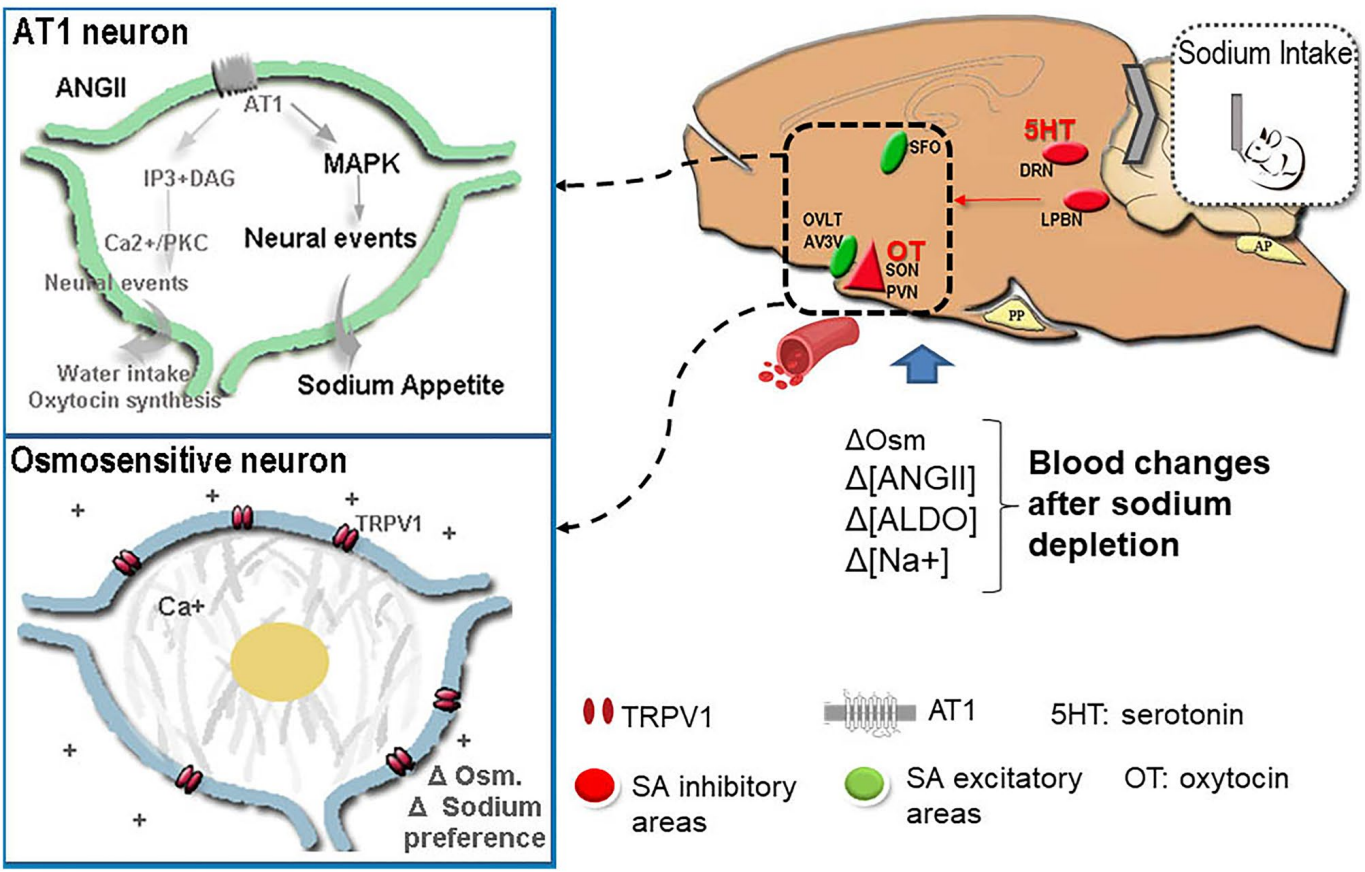
Schematic representation of different areas and mechanisms implicated in the regulation of sodium appetite after sodium depletion. *Panel of AT1 neuron:* AngII released after SD binds to the AT1 receptor activating the intracellular MAPK pathway to trigger sodium appetite. *Osmosensitive neuron panel:* changes in osmolarity, induced by SD, modify cell volume and the activity of TRPV1 channel that modulates sodium preference. DRN: dorsal raphe nucleus, LPBN: lateral parabrachial nucleus, SON: supraoptic nucleus, PVN: paraventricular nucleus, OVLT: oraganum vasculosum of the lamina terminalis; AV3V: anteroventral third ventricle area; SFO: subfornical organ, MAPK: mitogen-activated protein kinase, IP3: Inositol trisphosphate, DAG: diacylglycerol, AT1: angiotensinerigic receptor type 1, TRPV1: Transient receptor potential vanilloid subtype 1. Osm: Osmolarity, ANGII: Angiotensin II, ALDO: aldosterone.

The cerebral structures that control the excitatory appetitive and satiety phases of sodium intake are interconnected, constituting a neural network that orchestrates the sensory and integrative information response^3,7,8^ (Fig. 1). Previous evidence indicated that the modulation of salt appetite involves interactions between sensitive/receptive and excitatory vs. inhibitory nuclei and neuromodulator systems^9–11^. The excitatory circuit affects the subfornical organ (SFO) and the anteroventral third ventricle area (AV3V, including the organum vasculosum of the lamina terminalis (OVLT) among other nuclei), where angiotensin II (AngII) action is vital for the burst of sodium intake that follows a period of depletion^7,12–16^.

AngII binds the angiotensin II type 1 receptor (AT1) central receptor to induce thirst and sodium appetite, but this hormone recruits different intracellular players to modulate these behaviors^17–20^. SFO-AV3V AT1 activation takes the mitogen-activated protein kinase (MAPK) pathway, previously linked to AngII-induced increases in sodium appetite^17–26^. On the other hand, this excitatory pathway is typically limited by inhibitory hindbrain serotonergic (5-HT) and hypothalamic oxytocin (OXT) circuits^6,7,9,10,27–31^. We have detailed a specific 5-HT pathway [that includes the dorsal raphe nucleus (DRN, containing serotonergic neurons) and the lateral parabrachial nucleus (LPBN, the site of 5-HT action)] involved in the signaling of the satiation process of sodium-depletion-induced sodium appetite after sodium consumption that occurs 24 h after sodium depletion^6,10,11,27,31^. The OXT neural activity of supraoptic and paraventricular cells (SON and PVN, respectively) is implicated in the hypertonicity signaling after induced sodium intake (24 h after sodium depletion) since these are intrinsically osmosensors due to the presence of transient receptor potential channel 1 (TRPV1), like OVLT and SFO circumventricular organs (CVOs)^27,32^. Thus, as recently reported, TRPV1 KO mice have increased sodium preference after sodium depletion^33^.

Concerning the temporal dissociation between sodium depletion and the appearance of sodium appetite behavior, in previous studies, during the initial stage when sodium appetite is still inhibited (2 h after sodium depletion), we have demonstrated: 1- increased plasma renin activity and aldosterone concentration; 2- tonic 5-HT neural activity (as shown by Fos-5-HT immunoreactivity) along the DRN, which is involved in the inhibition of sodium appetite^6,31,34^; and 3- the involvement of LPBN serotonergic 2A and 2C receptors (5-HT_2A/2C_) in sodium appetite inhibition, as bilateral injections into the LPBN of the serotonin antagonist, methysergide, released the sodium appetite early when tested two h after sodium depletion compared to controls^6^. In contrast, 24 h after sodium depletion, when sodium appetite is evident, we observed: 1- decreased 5-HT-DRN neural activity; 2- plasma renin activity and aldosterone concentration remaining as elevated as it was two h after sodium depletion. These results suggest that 5-HT mechanisms in the DRN and LPBN block sodium intake, provoking the delay of sodium appetite even when the RAAS system is increased^6,34^.

In this work, we used real-time PCR in the DRN, LPBN, SFO, and AV3V to analyze mRNA the expression of AT1 (*Agtr1a),* 5HT_2C/2A_ *(Htr2c* and *Htr2a* respectively*)*, tryptophan hydroxylase enzyme (*Tph2*, enzyme of 5-HT synthesis), 5-HT transporter (*Slc6a4*), oxytocin receptor (*Oxtr*) and TRPV1 channel (*Trpv1)* genes, critical components of these circuits, at two h or 24 h after sodium depletion, coincident with the early inhibition and the later appearance of sodium appetite. Considering that the endogenous 5-HT receptor may be glycosylated for the membrane expression of the receptor^35–37^, we determined the temporal changes in the 5-HT_2C_ receptor along with the DRN, SFO and LPBN. We also analyzed Oxytocin coupling to neurophysin I (OXT-NP) by western blot and immunohistochemical detection of phosphorylated MAPK in the SON, PVN, SFO, and OVLT. Data were obtained at 2 or 24 h after sodium depletion. Given this data, we postulated the existence of temporary changes in the functioning of sensory, excitatory, and inhibitory components, or even in their intracellular pathways, which may occur to release sodium appetite after sodium depletion.

## Results

### Brain expression of Agtr1a mRNA after sodium depletion

**Figure 2:**
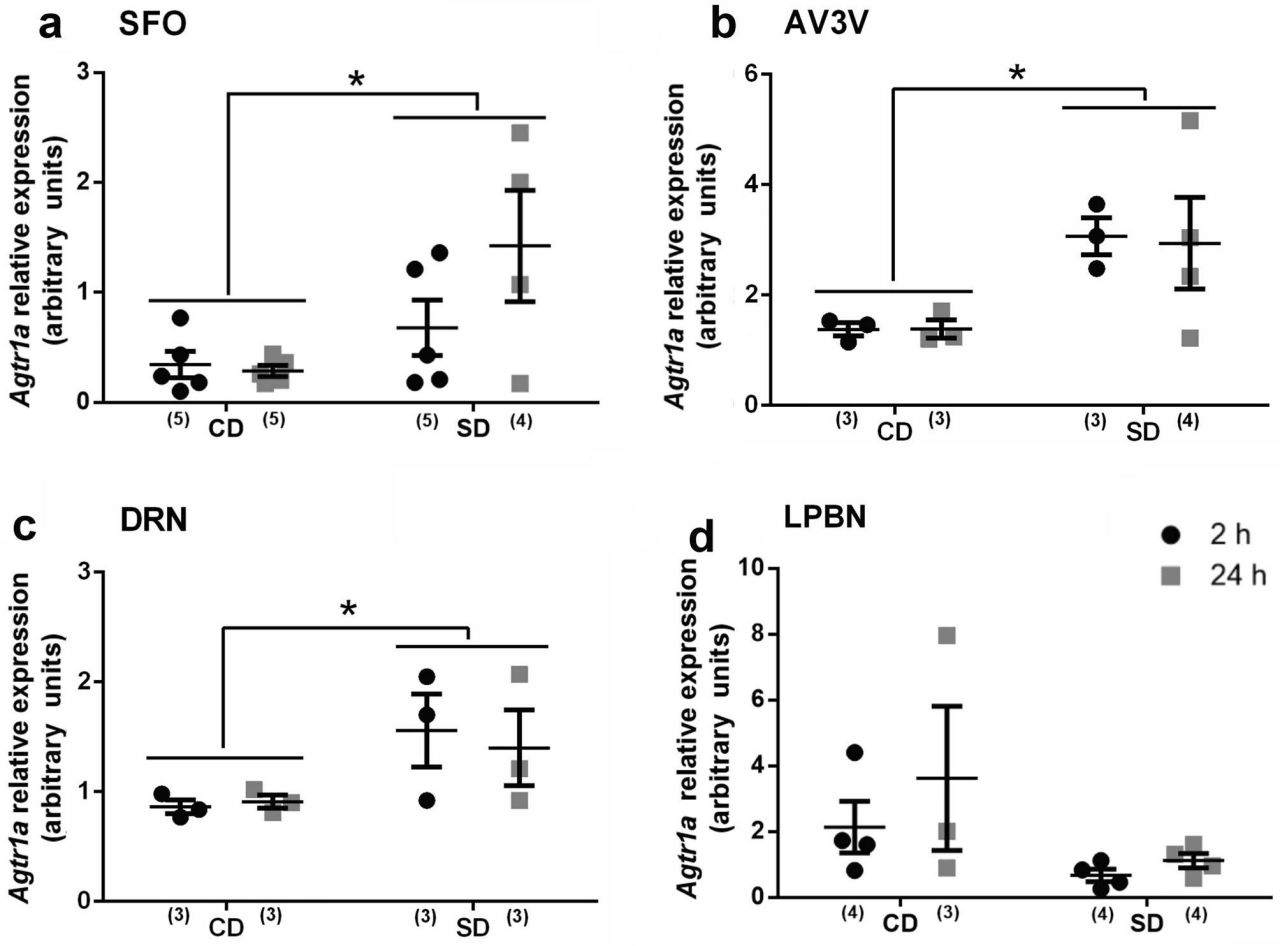
Brain Angiotensin 1a receptor expression after sodium depletion. Relative mRNA levels of Angiotensin 1a receptor (*Agtr1a*) in SFO (**a**), AV3V (**b**), DRN (**c**) and LPBN (**d**) at 2 h vs 24 h after sodium depletion. Values are mean ± SE (number of cases indicated by the individual points in each case). *p < 0.05 significant difference between SD versus CD. SD: Sodium-depleted group. CD: Control group. SFO: Subfornical Organ. AV3V: Anteroventral third ventricle region. DRN: Dorsal Raphe Nucleus. LPBN: Lateral Parabrachial Nucleus.

AngII has an excitatory effect on sodium appetite^8^, and previous results from our laboratory^6^ showed similar plasma renin activity and aldosterone concentration at two and 24 h after sodium depletion. Thus, we evaluated *Agtr1a* gene expression in regions involved in the excitatory and inhibitory control of sodium appetite. As shown in Fig. 2, there was a significant increase of *Agtr1a* mRNA along the DRN, SFO and AV3V after SD [sodium condition (CD vs. SD) main factor: (SFO: F_1.15_ = 8.12; *p* = 0.012; *η*2*p* = 0.35, Fig. 2a); (AV3V: F_1.9_ = 16.60; *p* = 0.0036; *η*2*p* = 0.64; Fig. 2b); (DRN: F_1.8_ = 8.15; *p* = 0.029; *η*2*p* = 0.42; Fig. 2c)]. It is important to note that the time after SD or the interaction between factors did not produce any significant effects in all these cases. We did not observe any significant difference in *Agtr1a* expression in the LPBN (Fig. 2d).

### Temporal pattern of phospho-ERK1/2 expression after sodium depletion

**Figure 3:**
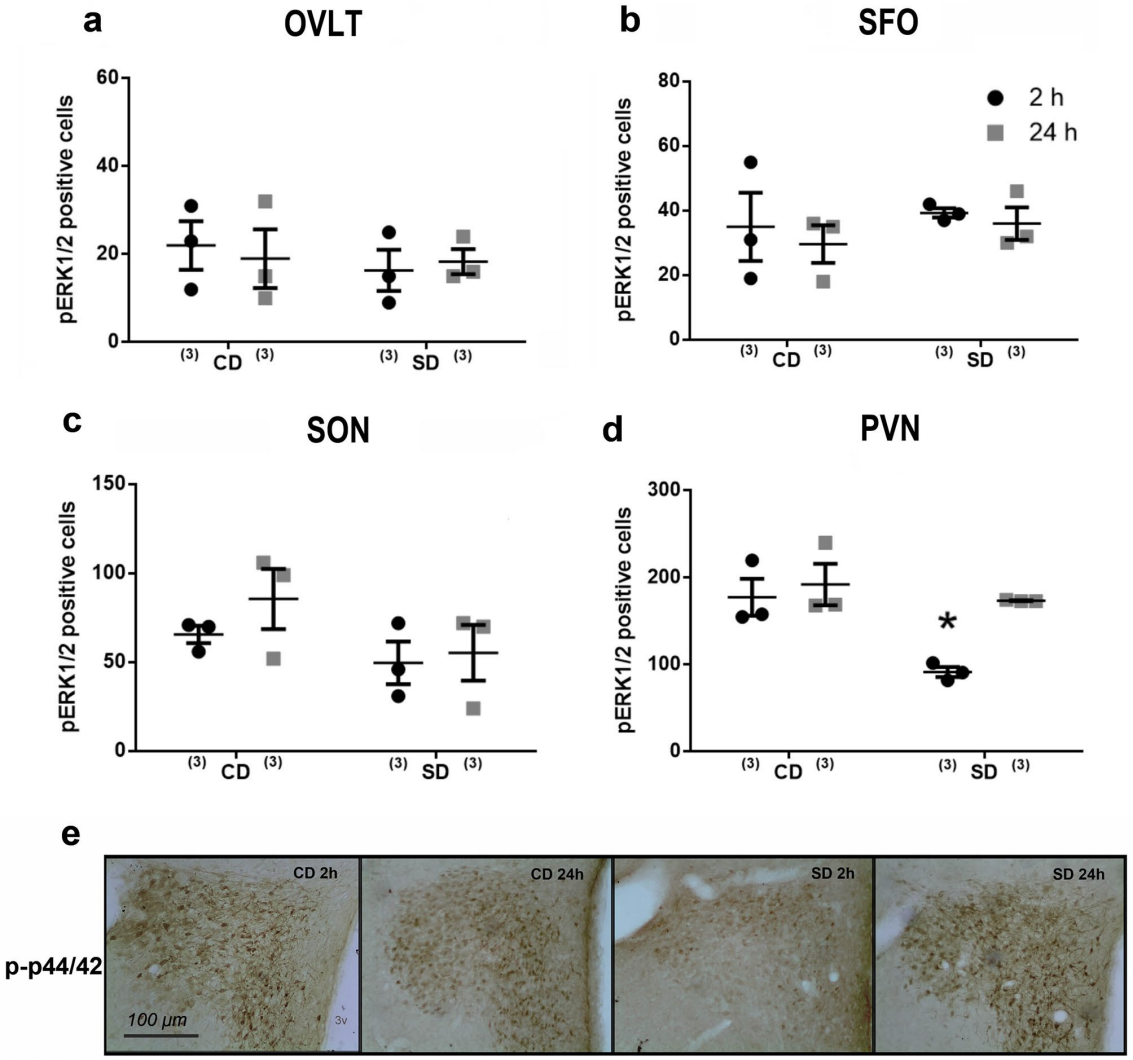
Brain pERK1/2 pattern after sodium depletion. The average number of pERK1/2 positive neurons in the OVLT (**a**), SFO (**b**), SON (**c**), and PVN (**d**) at two h and 24 h after sodium depletion. (**e**) Photomicrograph showing the pERK1/2 pattern of immunoreactive cells within the PVN. Scale bar = 100 μm. Values are mean ± SE (number of cases indicated by the individual points in each case). *p < 0.05 significant difference between SD two h versus other groups. SD: Sodium-depleted group. CD: Control group. OVLT: Organum Vasculosum Lateral Terminalis. SFO: Subfornical Organ SON: Supraoptic Nucleus. PVN: Paraventricular Nucleus.

The AngII-AT1 signaling pathways are involved in thirst and the onset of sodium appetite^17^. Thus, we used immunohistochemical analysis to assess the temporal pattern of ERK1/2 phosphorylation in the circumventricular organs of the lamina terminalis (SFO and OVLT) and hypothalamic areas (SON and PVN), implicated in the regulation of sodium appetite. We did not find significant temporal differences in ERK1/2 phosphorylation in the OVLT and SFO or the SON hypothalamic nucleus (Fig. 3a–c). Neither had we observed temporal changes in MAPK activity in separate experiments along the lateral margin and dorsal cap subdivision of OVLT (see Supplementary Fig. S1). However, along the PVN (at the medial level where ventral and medial parvocellular, dorsal cap, and lateral magnocellular subdivisions), we found a significantly different temporal effect (PVN: interaction: F_1.8_ = 6.11; *p* = 0.033; *η*2*p* = 0.42) (Fig. 3d and e). We observed a significant decrease in ERK phosphorylation at two hours compared to control values and those after 24 h after SD.

### Temporal brain-serotonergic system changes after sodium depletion

**Figure 4:**
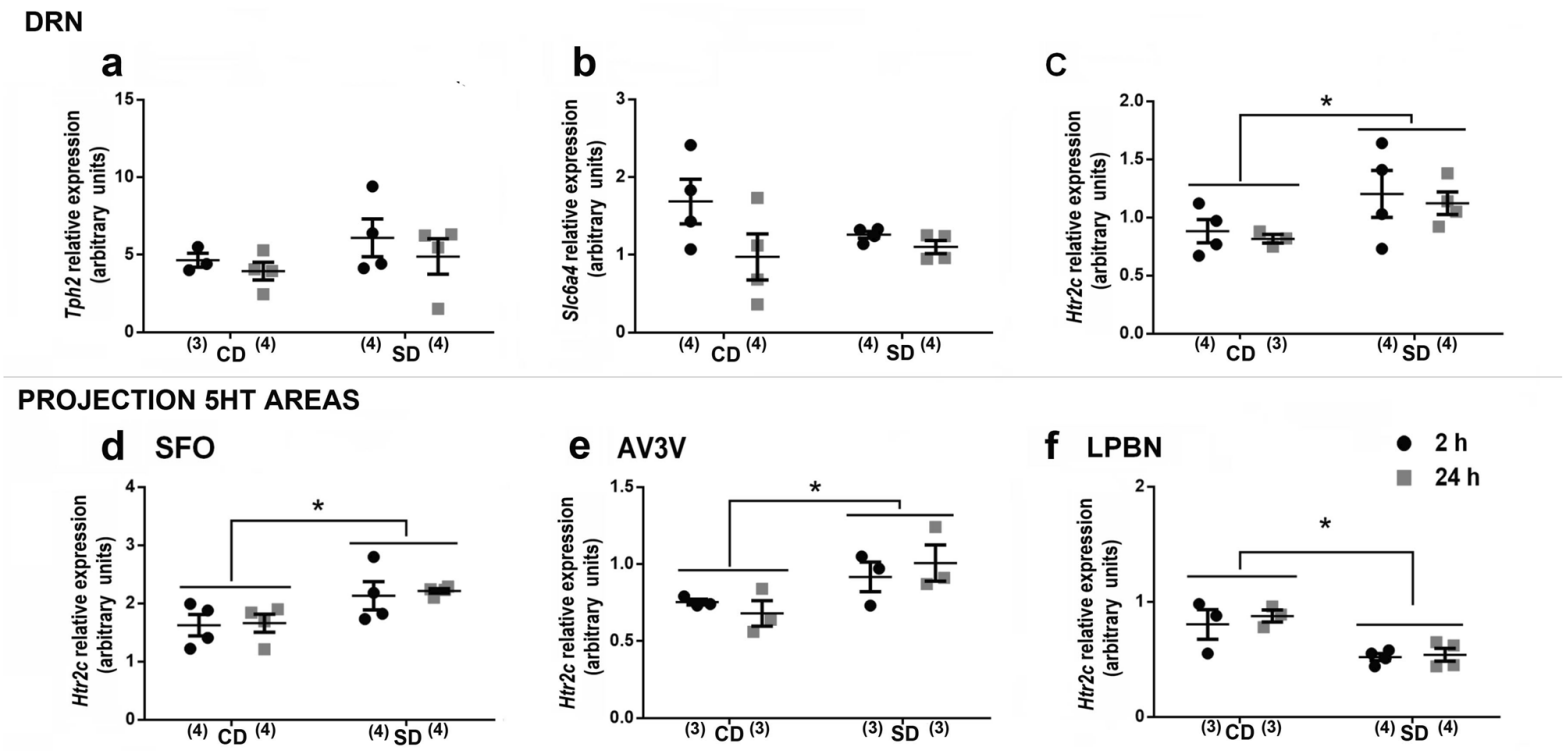
Brain serotonin system expression after sodium depletion. Relative mRNA level of tryptophan hydroxylase 2 (*Tph2*), Serotonin transporter (*Slc6a4*) and serotonin 2c receptors (*Htr2c*) in the DRN (**a**, **b** and **c**, respectively), and serotonin 2c receptors (*Htr2c*) in SFO (**d**), AV3V (**e**) and LPBN (**f**) at two and 24 h after sodium depletion. Values are mean ± SE (number of cases indicated by the individual points in each case). *p < 0.05 significant difference between SD vs. CD (**c** and **e**). SD: Sodium-depleted group. CD: Control group. DRN: Dorsal Raphe Nucleus. LPBN: Lateral Parabrachial Nucleus. SFO: Subfornical Organ. The relative mRNA levels of serotonin 2a receptor (*Htr2a*) in LPBN and SFO are presented in Supplementary Figure S2.

We also explored the temporal changes in the mRNA expression of *Tph2, Slc6a4, Htr2c and, Htr2a* along brain regions involved in sodium appetite control after SD. To analyze the local source of 5-HT, we determined the changes in gene expression for the 5-HT conversion enzyme, tryptophan hydroxylase-2 (*Tph2*) and the 5-HT transporter (*Slc6a4)* and the serotonin 2C (5-HT2C) receptors along the DRN, which was previously observed to be involved in the control of sodium appetite^27,30,38^. We observed no significant changes in either *Tph2* or *Slc6a4* mRNA expression along the DRN (Fig. 4a,b). However, we found a substantial increase in *Htr2c* mRNA expression in the DRN after SD (Sodium condition factor F_1.11_ = 5.41; *p* = 0.040; *η*2*p* = 0.33 Fig. 4c).

Considering the neuroanatomical and physiological 5-HT connection from the DRN to the SFO and AV3V, where sodium appetite is stimulated, and the LPBN, where sodium appetite is inhibited^6,10,11,43^, and the presence of serotonin 2A (5-HT_2A_) and 2C (5-HT_2C_) receptors in these areas^44^, we analyzed the temporal effect of SD on *Htr2c* and *Htr2a* expression. At the LPBN we observed a significant decrease in the expression of *Htr2c* mRNA after SD [sodium condition (CD vs SD) main effect: F_1.10_ = 18.29; *p* = 0.002; *η*2*p* = 0.44 Fig. 4f]. In contrast, we found a significant increase in *Htr2c* mRNA expression in the SFO and AV3V after SD (SFO: F_1.8_ = 28.07; *p* = 0.001; *η*2*p* = 0.67; AV3V: F _1,6_ = 14.80; *p* = 0.008; *η*2*p* = 0.50; Fig. 4d and e respectively). On the other hand, *Htr2a* mRNA expression in the LPBN and SFO did not change significantly after SD (See Supplementary Figure S2). The data indicate opposite expression patterns of Htr2c mRNA in the SFO/AV3V and the LPBN, in agreement with the antagonistic role of these areas in the physiological on sodium appetite.

### Temporal pattern of 5-HT_2C_ receptor glycosylation after SD

**Figure 5:**
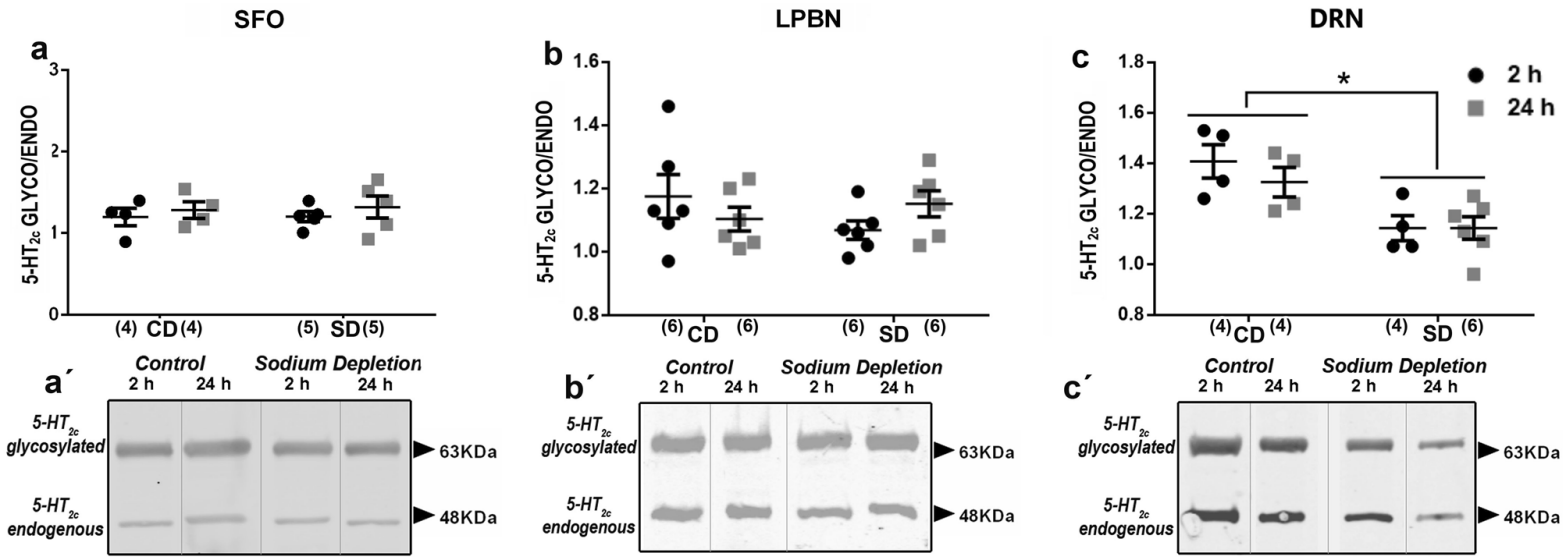
Glycosylation of 5HT2c receptors after sodium depletion. 5-HT_2C_ receptor glycosylate/endogenous relationship in SFO (**a**), LPBN (**b**), DRN (**c**) at two and 24 h after sodium depletion. Western blot detection of glycosylated and endogenous 5HT_2C_ receptor protein expressed in SFO (**a’**), LPBN (**b’**) and DRN (**c´**). Values are mean ± SE (number of cases indicated by the individual points in each case). *p < 0.05 significant difference between SD versus CD. SD: Sodium-depleted group. CD: Control group. SFO: Subfornical Organ. LPBN: Lateral Parabrachial Nucleus. DRN: dorsal raphe nucleus. Blots: Dividing grey lines indicated non-contiguous lanes from different parts of the same gel. Full-length blots are presented in Supplementary Figure S3.

Considering the antagonism in the control of sodium appetite by the LPBN and SFO nuclei, we also analyzed the regulation of 5-HT_2C_ glycosylation in these areas. However, the LPBN and SFO showed no glycosylated/endogenous 5-HT_2C_ ratio changes (Fig. 5a and b). We also analyzed the regulation of 5-HT_2C_ glycosylation in the DRN. We observed that the ratio of glycosylated/endogenous 5-HT_2C_ in the DRN decreased after SD (F_1.12_ = 13.08; p = 0.003; *η*2*p* = 0.33 Fig. 5c). Together the above results suggest an influence of changes in natremia in the expression and localization of 5-HT_2c_-LPBN-SFO-DRN components, adjusting the ability of these areas to respond during a body sodium challenge.

### Temporal changes in the brain-oxytocin system after SD

**Figure 6:**
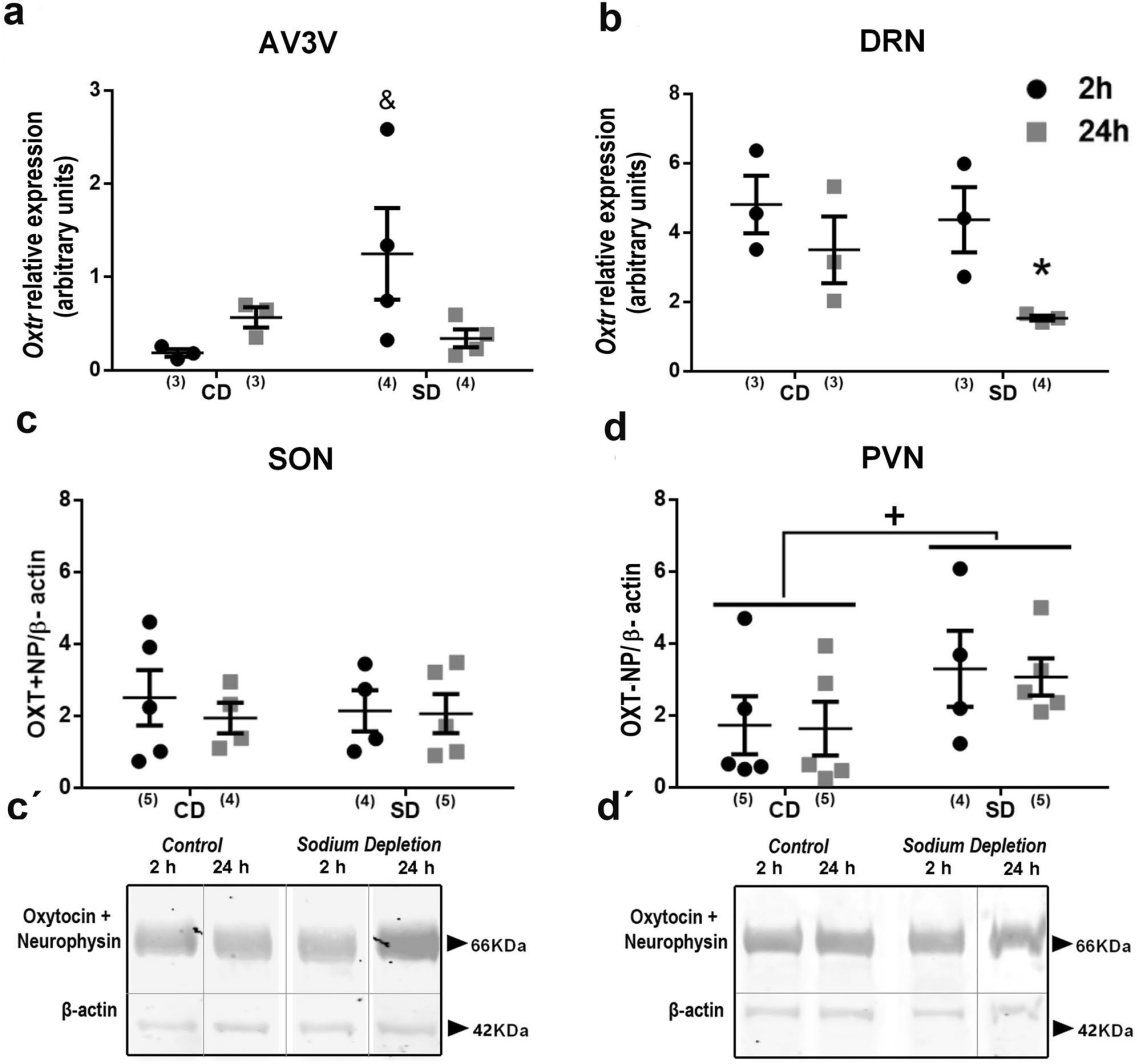
Brain oxytocin system expression after sodium depletion. Relative mRNA levels of oxytocin receptor (*Oxtr*) in the AV3V (**a**) and DRN (**b**) and relative protein expression of Oxytocin + neurophysin (OXT-NP) in the SON (**c**) and PVN (**d**) at two and 24 h after sodium depletion. Western blot detection of OXT-NP protein expressed in SON(c') and PVN(d'). Values are mean ± SE (number of cases indicated by the individual points in each case). (**a**) & *p* < 0.05 significant difference between SD two h vs. other groups. (**b**) *p < 0.05 significant difference between SD 24 h vs. other groups. (**d**) + *p* < 0.05 significant difference between CD vs. SD. SD: Sodium-depleted group. CD: Control group. AV3V: Anteroventral third ventricle area. DRN: Dorsal Raphe Nucleus. PVN: Paraventricular nucleus. SON: Supraoptic Nucleus. Blots: Dividing grey lines indicated non-contiguous lanes from different parts of the same gel. Full-length blots are presented in Supplementary Figure S4.

Central Oxytocin inhibits SA through its receptors (*Oxtr*) localized in different critical structures such as the DRN and the AV3V, where PVN-efferences arrive^46^. Determining changes in components of the oxytocin system enabled us to analyze the postulated inhibitory role of the oxytocinergic system on sodium appetite^9,27,45^. We observed a significant decrease of Oxytocin receptor (*Oxtr*) gene expression along the DRN at 24 h after SD when sodium appetite appeared (interaction F_1.6_ = 122.41; *p* < 0.001, η2p = 0.45; Fig. 6b). In contrast, *Oxtr* was significantly increased two hours after SD in the AV3V (interaction F_1.9_ = 6.00; *p* = 0.032; *η*2*p* = 0.39), in agreement with an early inhibition of sodium appetite after SD (Fig. 6a,b). We also analyzed the temporal oxytocin-neurophysin (OXT-NP) content along with the SON and PVN. As Fig. 6c and d show we did not find any significant differences in the SON, but a considerable increase at the PVN after SD regardless of time (sodium condition: F_1.16_ = 11.09; *p* = 0.005; η2p = 0.20) (Fig. 6d).

### Temporal changes in Trpv1 mRNA expression at the AV3V and SFO after SD

**Figure 7:**
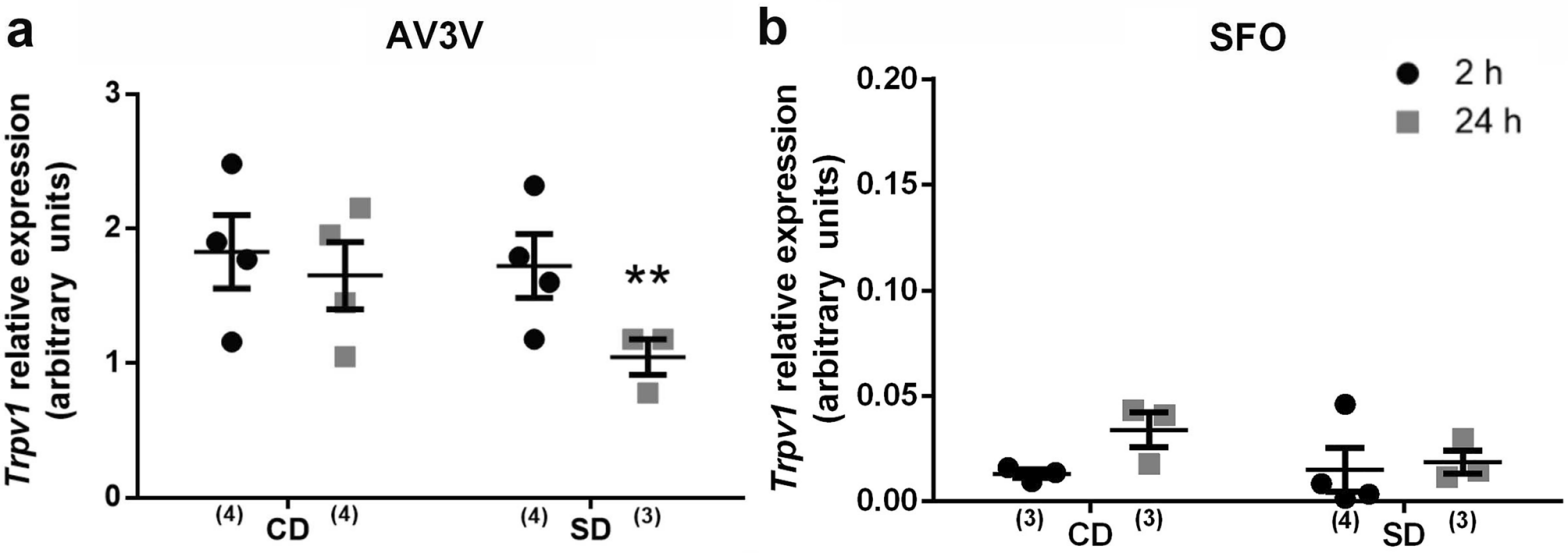
Brain *Trpv1* mRNA expression after sodium depletion. Relative mRNA levels of transient receptor potential vanilloid type 1 (*Trpv1*) in AV3V (**a**) an SFO (**b**) at two and 24 h after sodium depletion. Values are mean ± SE (number of cases indicated by the individual points in each case). **p < 0.05 significant difference between SD 24 h versus other groups. SD: Sodium-depleted group. CD: Control group. AV3V: Anteroventral third ventricle.

Our recent results showed that the TRPV1 channel, implicated in central osmosensation, is essential to the sodium preference induced by SD^33^. Osmosensation in response to changes in sodium balance is detected by the CVOs of the lamina terminalis, including SFO and OVLT, and the Oxytocin or vasopressin magnocellular cells, whose osmosensitivity relies on the presence of this channel^32^. Thus, we explored TRPV1 channel expression at two and 24 h after SD along the AV3V and SFO. As shown in Fig. 7a, we found a significant decrease 24 h after SD in Trpv1 mRNA expression in the AV3V (interaction F_1.8_ = 6.22; *p* < 0.037; *η*2*p* = 0.17), Fig. 7a. However, *Trpv1* expression along the SFO did not change significantly after SD, Fig. 7b.

## Discussion

Knowledge about thirst and sodium appetite's cellular and molecular mechanisms, two physiologically important behaviors, has grown enormously during recent decades^17,20,42,49^. This accumulated evidence opens a new chapter in our understanding of hydroelectrolytic homeostasis. The present study provides further information on the temporal behavior of critical players controlling sodium appetite.

**Figure 8:**
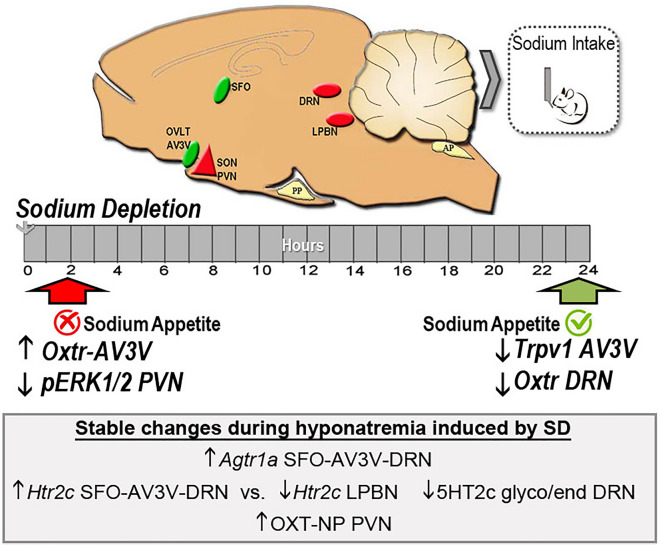
Multi-system, integrated responses after sodium depletion. After sodium depletion the expression of critical components within the brain control circuit of sodium appetite, including Angiotensin-type-1 receptor, Oxytocin-neurophysin-I, and serotonergic-(5HT)-type-2c receptor were changed regardless of time (grey box). When sodium appetite is inapparent, at two hours after SD, there is reduced MAPK phosphorylation in the PVN and increased Oxtr mRNA expression in the AV3V. At twenty-four hours after SD, when sodium appetite is released, there is a reduction of Trpv1 and Oxtr mRNA expression in the AV3V and dorsal raphe nucleus, respectively. The results indicate that SD exerts a plastic temporally coordinated effect to promote the onset of sodium appetite and restore water-electrolyte homeostasis. DRN: dorsal raphe nucleus, LPBN: lateral parabrachial nucleus, SON: supraoptic nucleus, PVN: paraventricular nucleus, OVLT: oraganum vasculosum of the lamina terminalis; AV3V: anteroventral third ventricle area; SFO: subfornical organ, pERK: phosphorilated extracellular signal-regulated kinase, Agtr1a: angiotensinerigic receptor type 1 gene, Trpv1: Transient receptor potential vanilloid subtype 1 gene. Oxtr: oxytocin receptor gene, Htr2c: serotonergic receptor type 2c gene, OXT-NP: Oxytocin + neurophysin, 5HT2C glyco/endo: glycosilated serotonergic receptor type 2c.

The appearance of sodium appetite delays several hours after body sodium deficiency. However, the humoral stimulatory signals as hyponatremia, osmolality, and Angiotensin II emerge immediately after SD. These signals are centrally detected by the circumventricular organs of the lamina terminalis, which features different sensors, channels, and receptors. This information is organized and integrated into excitatory and inhibitory brain areas to trigger the sodium appetite gradually (Fig. 8). Each brain structure presents different temporal patterns of adaptation to provide a proper response. For example, in the SFO of mice, we observed an increase in TRPV4 expression during the first hours after SD^32,33^. Still, later, when SA appeared, the NaX channel expression decreased (involved in the hypernatremic detection)^33,63^. In the present study, we demonstrated that the expression of TRPV1 channel mRNA along the AV3V decreased 24 h after SD, suggesting a change in the osmotic threshold that allows the hypertonic sodium consumption during the sodium appetitive phase. According to this, TRPV1-/- mice (hyperosmotic sensor) heightened sodium preference after SD^33^. AT1 receptor activation by AngII has a dual effect, on the one hand, it increases water and salt intake after SD (at CVOs of the lamina terminalis level), and on the other, it stimulates Oxytocin synthesis at the hypothalamic level, which is a sodium intake inhibitor system^18,19,54,55^. Our results indicate that SD elicits an increase in the expression of the AT1 receptor at the SFO, AV3V, and DRN levels. Likewise, evidence shows that the binding of ANGII to the AT1R activates different intracellular signaling pathways to stimulate thirst and appetite for sodium, being ERK phosphorylation fundamental for triggering SA. Although we did not observe an increase in ERK phosphorylation at 24 h after SD, we observed a decrease at the PVN at 2 h after SD, which coincides with the early inhibition of sodium appetite. The present results confirmed a tonic SD-induced increase in OT synthesis at the PVN level due to AngII increases without any temporal changes. However, the inhibitory effect of OXT on SA occurs after binding to its receptor. Our results also show an early rise in OXTR gene expression in the AV3V and a late decrease at the DRN level, suggesting an early inhibitory effect on excitatory areas of SA and a late modulatory impact at the DRN level (possibly on 5HT neurons). Finally, the 5HT system did not present any temporal changes in its components in the areas analyzed.

Interestingly, however, gene expression of the 5HT_2C_ receptor was opposite in contrast to functional areas on SA, such as SFO and AV3V vs. LPBN. Likewise, glycosylation of the 5HT_2C_ in the DRN decreased after SD. This posttranslational changes in this structure may form part of a rapid compensatory mechanism to stop sodium consumption during the body sodium reestablishment (Fig. 8).

Numerous studies have shown that the genesis of thirst and SA involve both the peripheral and the central RAAS^47–50^. Lesions of either the SFO or the AV3V impair angiotensin-induced sodium intake induced by SD^12,14–16,51,52^. AngII binds to AT1 in the CVOs of the lamina terminalis, specifically the SFO and the OVLT included in the AV3V region. The SFO, containing many AT1 receptors, is the target organ for AngII^44,49^. SFO-AngII infusion increased cell activity by 70% and decreased local 5-HT release^53,54^. In agreement with these results, we found an increased expression of *Agtr1a* after SD in the SFO and AV3V excitatory areas but increased in the DRN. These results are also in agreement with studies where dietary and pharmacological SD by furosemide increase both *Agtr1a* mRNA levels (by in situ hybridization) and AT1 binding sites (by autoradiography) in the SFO and the DRN^38,47,50^. These results suggest that an increase in AT1 receptor favors an effect of AngII in the induction of SA.

In this regard, AngII binding to the AT1 stimulates the central AngII system, activating its intracellular pathways implicated in both thirst and induction of SA, specifically involving MAPK signaling in sodium appetite onset^17–20^. The rapid pharmacological model of sodium appetite stimulation (FURO + CAP) increased MAPK activity along the CVOs of the lamina terminalis and the PVN/SON hypothalamic areas^18^. In contrast to these data, our results, using furosemide combined with a low sodium diet protocol, showed that MAPK phosphorylation did not change along the CVOs of the lamina terminalis. However, we observed a decrease of MAPK activity at two hours after SD in the PVN during the early inhibition of sodium appetite. On the other hand, several studies^18,19,54^ demonstrated that a signaling pathway different from MAPKs involved OXT neuron stimulation induced by SD or AngII. This finding may indicate that during the early inhibition of SA observed in our model, AngII stimulates another signaling cascade, such as PKC, exciting the oxytocin system that inhibits SA. In line with this hypothesis, we also observed an increase of OXT-NP in the PVN at two and 24 h after SD.

Several studies postulated an inhibitory role of 5-HT on sodium appetite, with the LPBN as the main structure involved in this effect^6,11,27–29,31,34,58^. The pharmacological antagonism of 5-HT_2C/2A_ in the LPBN increases SA even as early as two hours after SD when SA is inapparent^6,29^. A recent study^42^ demonstrated that the 5-HT_2C_ cluster of neurons within the LPBN participated in the regulation of sodium intake. Its tonic activity inhibits SA under hypo- and euvolemic states, providing the source of 5-HT from the raphe projections (dorsal and median subdivisions). The present study suggests a decrease in *Htr2c* expression along the LPBN during hyponatremia, without any temporal relation to the onset of SA. Thus, the changes in the chronically released serotonin at LPBN after SD may be the crucial step to the tonic SA inhibition, as was previously demonstrated by microdialysis^65^. Our previous work also indicates that 5HT levels decrease in the DRN during sodium depletion, and the activity of 5HT neurons increases upon body sodium overload^31,38^. The present data indicate that, in this area, sodium depletion modifies both the expression and the glycosylation pattern of the 5HT_2C_ receptor at the DRN level, but not the expression of the enzyme synthesis nor the 5HT transporter. These data lead us to propose that the 5HT_2C_ receptor at the DRN level could be a mechanism involved in regulating serotonin activity and synthesis in response to changes in body sodium status.

Takahashi and Tanaka^43^ showed that the release of 5-HT into the SFO significantly decreased during SA and increased during the satiety phase (after sodium consumption induced by sodium depletion), implying a tonic inhibitory control of SA in this area. The SFO, where AngII normally stimulates SA, also presents 5-HT_2A/2C_ receptors^44^. Here we observed an increased *Htr2c* mRNA expression at this level, suggesting a possible anticipatory effect to avoid overconsumption while reestablishing sodium balance by sodium intake.

The "disinhibition hypothesis" postulates that excitatory systems of SA are active after SD and are held in check by inhibitory mechanisms that gradually stop to allow the appearance of SA^55^. Previous results suggested that the oxytocin system is associated with inhibiting SA^27,45,56^. Our present results show that the OXT-NP system at the PVN rapidly increases after two hours of SD. However, its receptor has a temporal behavior at the AV3V, increasing two hours after but returning its expression 24 h after SD, which may slow down and later allow sodium consumption. We also observed a decrease of *Oxtr* expression 24 h after SD at the DRN (the primary source of 5-HT neurons), possibly modulating the activity of serotonergic neurons during SA or after sodium consumption, as we previously observed^6,27,31,34^. Likewise, there is evidence that both the serotonin and oxytocin systems switch each other in the brain^57,58^.

The central TRPV1 channel has been implicated in osmosensation and thermoregulation^32,59,60^. This protein is expressed in critical structures associated with the control of hydroelectrolytic homeostasis, including the CVOs of the lamina terminalis (SFO and OVLT) and magnocellular oxytocin- and vasopressin-cells. Our previous results indicated that TRPV1 knock-out mice have an increased sodium preference after SD, suggesting an inhibitory role in modulating SA^33^. Our present results also show that the AV3V region significantly decreases *Trpv1* expression 24 h after SD, coincident with the appearance of SA. However, we could not rule out the participation of the SON, which is intrinsically osmosensitive because of the presence of TRPV1 in this effect, as the punch technique to the collection of the samples also included the rostral part of this nucleus. The area analyzed contains the OVLT, the main central osmosensor^60,61^, and part of the SON with the magnocellular cells, which enable the regulation of the release of OXT and AVP. Our recent study also showed that TRPV1 KO mice had lower Fos immunoreactivity, suggesting reduced activity after SD in the OVLT and AVP-SON neurons^33^. In our model of SD, we have an early and continuous increment in AngII that is possibly involved in OXT synthesis stimulation, as we previously observed^19^. However, the AngII induced by SD concurs with hypoosmolality/hyponatremia. Chakfe and Bourque^62^ showed that AngII could increase the osmosensitive gain in magnocellular cells, which agrees with our data. Still, the authors also found that the AngII-stimulated channels inactivate by hyponatremia, which increases membrane stretch. Summing up, SD provides at least two physical–chemical forces, hypoosmolality/hyponatremia and AngII release, which modulate TRPV1 activity in opposite ways; however, SD also reduces temporally *Trpv1* expression in the AV3V. The reduction in TRPV1 activity possibly decreases the capacity for hypertonic sensation, allowing the entry of hypertonic sodium solution to the organism during SA.

In summary, the present results indicate that RAAS activation induced by SD has two effects, promoting salt intake through changes in MAPK phosphorylation and preventing future overconsumption by OXT changes. At the DRN, SD also decreased the glycosylated form of the 5-HT2cR. The *Htr2c* receptor also has opposite expression patterns at the SFO/AV3V and LPBN, matching its role in inhibiting and stimulating SA. Finally, the early increase of *Oxtr* and the later decrease in *Trpv1* expression in the AV3V may temporally affect the appearance of SA. Thus, the physiologically significant behavior, SA, is modulated by an underlying complex brain circuit involving different components in each area, which coordinate their responses over time to reestablish hydroelectrolytic homeostasis.

## Materials and methods

### Animals

For the experiments, we used adult male Wistar rats, born and reared in the breeding colony at Instituto Ferreyra (INIMEC-CONICET-UNC, Córdoba, Argentina). Animals weighing 250–300 g were housed singly in metabolic cages with free access to a normal sodium diet (Purina Rat Chow), distilled water, and hypertonic solution (NaCl 2%) for three days of adaptation^6^. Room lights were on for 12 h/day kept at 23ºC. All experimental protocols were approved by INIMEC's animal care and use committee under protocol # 016/2021, following the guidelines of the international Public Health Service Guide for the Care and Use of Laboratory Animals (NIH Publications No. 8023, revised 1978). We complied with the ARRIVE guidelines.

### Sodium depletion protocol

The experimental animals received a subcutaneous injection of furosemide (20 mg/kg, Lasix, Sanofi-Aventis Pharma, Brazil). The isotonic saline injection controls immediately before the transfer to clean individual metabolic cages, as previously described^10,38^. The experimental group (*n* = 30) had immediate access to distilled water and low sodium food (ICN, Costa Mesa, CA, USA, sodium content 0.002%) for the next 2 and 24 h. Control rats (*n* = 30) were subjected to a similar procedure but had access to filtered water and a standard rodent diet (sodium content 0.2%). At the end of 2 and 24 h, rats were decapitated for mRNA and protein determinations (48 rats) or perfused transcardially for immunohistochemistry procedure (12 rats). To confirm the hyponatremic effect of Furosemide treatment, we determined SD-induced changes in Na + , Cl− and protein concentration and osmolality in blood samples. Trunk blood was collected in plastic tubes containing EDTA (final concentration 2 mg/ml blood) and immediately centrifuged at 4 °C for 20 min at 3.000 g. Then plasma was removed and kept at − 20 °C until determination. Plasma sodium and chloride concentrations were determined using an Ion-Selective Electrode (Hitachi Modular P + ISE. Roche 8 Diagnostic). Plasma osmolality was analyzed by vapor pressure osmometry (VAPRO 5520), and plasma volume was indirectly inferred by the plasma protein concentration, measured in an absorbance microplate reader (BioTek EL800) according to the protocol proposed by Lowry et al. (1951). (See Supplementary material table S1).

### Relative mRNA expression in brain areas

Immediately after decapitation, the brains were collected and frozen in dry ice in RNAse-free conditions and stored at − 80 °C for *Agtr1a, Oxtr, Htr2c, Htr2a, Trpv1, Tph2, Slc6a4,* and *Gapdh* mRNA determinations by qPCR assay. Coronal sections of 1320 μm for the dorsal raphe nucleus (DRN; bregma: − 7.3 to − 8.2 mm), 1380 μm for the lateral parabrachial nucleus (LPBN; bregma: − 8.7 to − 9.8 mm), 1320 μm for the subfornical organ (SFO; bregma: − 0.8 to − 1.4 mm), and 1320 μm for the anteroventral third ventricle region (AV3V; bregma: − 0.8 to − 1.4 mm), were obtained from the frozen brains in a microtome with stainless-steel needle punches of two different diameters (inner diameter 1.5 mm to SFO and LPBN; inner diameter 2 mm to AV3V and DRN). The AV3V comprises several distinct brain areas, including the preoptic periventricular region, the ventral part of the median preoptic nucleus, the anterior hypothalamic periventricular area the organum vasculosum of the lamina terminalis (OVLT). Due to the size of our punch, the sample also included the rostral part of the supraoptic nucleus. According to a rat brain atlas, the brain nuclei were identified and delimited^39^. RNA was isolated from punched brain tissue using Trizol reagent (Invitrogen, Carlsbad, CA, USA) as directed by the manufacturer with some modifications: RNA precipitation with isopropanol was performed overnight at − 20 °C. DNase-treated (Fermentas) RNA was quantified using a NanoDrop 2000 UV–Vis spectrophotometer and was then reverse-transcribed into cDNA (enzyme RTM-MLV—Promega). Brain *Gapdh*, *Agtr1a, Oxtr, Htr2a, Htr2c, Trpv1, Tph2*, and *Slc6a4* gene expression was determined using Syber Green Real-Time PCR Master Mixes (Applied Biosystems™) in the Step One Real-Time equipment (Applied Biosystems). Primer sequences are in Table 1.

**Table 1:** Primers pairs for *Gapdh, Agtr1a, Oxtr, Htr2a/2c,Tph2, Slc6a4* and *Trpv1.*

*Gene*	GenBank access number	Forward Primer 5ʹ-3ʹ	Reverse Primer 5ʹ-3ʹ	Product lenght (bp)	Annealing temp. (°C)
*Gapdh*	NM_017008.4	TGTGAACGGATTTGGCCGTA	ATGAAGGGGTCGTTGATGGC	93	60
*Agtr1a*	NM_030985.4	AACCCTCTGTTCTACGGC	ACCTGTCACTCCACCTCA	194	56.5
*Oxtr*	NM_012871.3	CGTACTGGCCTTCATCGTGT	GAAGGCAGAAGCTTCCTTGG	94	59.5
*Htr2c (5Ht*_*2C*_*)*	NM_012765.3	TTGGACTGAGGGACGAAAGC	GGATGAAGAATGCCACGAAGG	102	59.6
*Htr2a (5Ht*_*2A*_*)*	NM_017254.1	AACGGTCCATCCACAGAG	AACAGGAAGAACACGATGC	109	56
*Tph2*	NM_173839.2	CACCTGACAACATTTGGACG	ACGTTGTCCTTGAATCCTGG	148	57.6
*Slc6a4 (SerT)*	NM_013034.4	GAACTCCTGGAACACTGGCA	CAGGACATGGCGCAAGTAGA	109	60
*Trpv1*	NM_031982.1	TTCACCGAATGGGCCTATGG	TGACGGTTAGGGGTCTCACT	125	59.9

### Calculations of relative gene expression

The relative quantification was determined by the ΔΔCt method, where the fold change of mRNA content in the unknown sample relative to the control group was determined by 2 ^-ΔΔCt^ (ΔΔCt = ΔCt _unknown_ − ΔCt _control_). For each sample, the Ct was determined and normalized to the average of the housekeeping *Gapdh*. This gene is a constitutive and stable gene between groups, which allows its use as a control for this experiment. All samples were run in duplicate with the average CT used for each sample. The Ct of the calibrator group (the mean Ct of the naïve male adult rat) was then subtracted from each sample to give a Ct value. Relative quantification of the *Agtr1a, Oxtr, Htr2a, Htr2c, Trpv1, Tph2, and Slc6a4* gene expression was normalized to the naïve male adult rat. Data are presented as mRNA relative to the control calibrator group.

### Western blot analysis

Animals were killed by decapitation. Coronal sections of 1380 μm for the LPBN (bregma: − 8.7 to − 9.8 mm), 1320 μm for the SFO (bregma: − 0.8 to − 1.4 mm), 1000 μm for the paraventricular nucleus (PVN; bregma: − 1.32 mm to − 2.04), and 1200 μm for the SON (bregma: − 0.8 to − 1.8 mm) were obtained from the frozen brains in a microtome with a stainless-steel punch needle (inner diameter 1.5 mm). The brain nuclei were identified and delimited according to Paxinos and Watson^39^, and were homogenized in radio-immunoprecipitation assay (RIPA) buffer containing protease and phosphatase inhibitors (phenylmethylsulfonyl fluoride 100 μg/ml; leupeptin 1 μg/ml; pepstatin 10 μM 1 μg/ml; aprotinin 1 μg/ml; sodium orthovanadate 1 mM; chemostatin 5 μg/ml; antipain 5 μg/ml; NaF 50 mM; phenanthroline 1 mM and sodium pyrophosphate 10 μM). The homogenates were clarified by centrifugation at 13,000 rpm at 4 °C for 7 min in the cold. The protein concentration was determined using the Lowry method^40^. Samples were then boiled in gel-loading buffer and separated using sodium dodecyl sulfate–polyacrylamide gel electrophoresis (SDS-PAGE, 10%). Thirty micrograms of total proteins were loaded in each line. Proteins were transferred to a 0.2 μm nitrocellulose membrane (Bio-Rad Laboratories Inc., PA, USA) and blocked with 5% non-fat milk in Tris-buffered saline with 0.05% Tween for one h at room temperature (RT). Membranes were then incubated overnight at 4 °C with the primary antibody of interest: mouse monoclonal antibodies to serotonin 2C receptors (Santa Cruz Biotechnology Cat# sc-17797; dilution:1:100) oxytocin-neurophysin I (OXT- NP. Antibody PS 38 procedures by Ben-Barak et al.^41^; dilution 1:25 kindly donated by Dr. Harold Gainer (NIH, Bethesda, USA). Then the membranes were incubated with the anti-mouse fluorescent secondary antibody (dilution 1:5000—LI-COR Biosciences Cat# 926–32222) for one hour at room temperature with shaking. The membrane was then imaged by fluorescence in the infrared range using an Odyssey scanner (LI-COR Biosciences). Membranes were re-probed with the rabbit polyclonal antibody, beta-Actin (PA1-46296) (Thermo Fisher Scientific Cat# PA1-46296, dilution 1:1500), to control protein loading. The membranes were then incubated with the anti-rabbit fluorescent secondary antibody (dilution 1:5000—LI-COR Biosciences Cat# 926–32213) for one hour at room temperature with shaking. The membrane was imaged by fluorescence in the infrared range using an Odyssey scanner (LI-COR Biosciences). For the antibodies, validation is provided on the manufacturer’s website and OTX-NP by Ben-Barak et al.^41^. Band intensities were quantified with NIH Image J software (National Institutes of Health, Bethesda, MD, 66, Fiji (RRID:SCR_002285); https://fiji.sc/). The total content of beta-actin normalized the proteins.

### Immunohistochemistry

Rats were anesthetized with thiopentone (100 mg kg‐1 i.p.) and perfused transcardially with 100 mL of 0.9% saline solution, followed by 400 mL of 4% paraformaldehyde in 0.1 mol L‐1 phosphate buffer (PB) (pH 7.2). Brains were removed fixed in the paraformaldehyde solution by overnight incubation. The brains were stored at 4 °C in PB containing 30% sucrose until processing when coronal sections of 40 µm were cut using a freezing microtome. The sections were incubated for 48 h at 4 °C with polyclonal rabbit Phospho p44/42 MAPK (phosphorylated extracellular signal-regulated kinase, pErk1/2) antibody (Cell Signaling Technology Cat# 4376, dilution: 1:2000).

Afterward, the sections were rinsed and incubated for one h at room temperature with biotin-labeled anti-rabbit immunoglobulin and an avidin–biotin-peroxidase complex. Cytoplasmic phospho p44/42 MAPK-it was detected with unintensified DAB, which produces a brown reaction product. The brain nuclei exhibiting pERK1/2 were identified and delimited according to Paxinos and Watson^39^. The distance from the bregma of the corresponding plates was as follows: OVLT: 0.50 mm, SFO: − 0.90 mm, PVN: − 1.60 mm, and SON: − 1 mm. Cytoplasmic pERK1/2-ir was quantified with a computerized system that included a Zeiss microscope with a DC 200 Leica digital camera attached to a contrast enhancement device. Images were digitalized and analyzed using NIH Image J software (National Institutes of Health, Bethesda, MD,66, Fiji (RRID:SCR_002285); https://fiji.sc/). Representative sections of the different groups were acquired at approximately the same plate level, with the Adobe Photoshop Image Analysis Program, version 7.0. We counted the number of pERK1/2-ir cells at one level for each analyzed structure using an equal defined area for all images corresponding to the same nucleus^64^. The counting was done on three animals in each group and repeated at least twice on each section to ensure that the profiles' numbers were similar. Areas were analyzed by an experimenter blinded to the experimental groups.

### Statistical analysis

All data are expressed as mean ± standard error (SE). The normality of the data was assessed with the Shapiro–Wilk test (Supplementary table S2). All variables were analyzed by appropriate two-way analyses of variance (ANOVA) (sodium condition and time after SD as main factors). For real-time PCR results, we used a two-way ANOVA in randomized blocks. Statistical significance was established at *p* < 0.05. Analyses were performed using InfoStat, (RRID: SCR_014310). Statistically significant interactions were further analyzed using the Tukey test (type I error probability was set at 0.05). The partial eta-squared (*η*2*p*) was used to describe effect sizes of the ANOVAs, and was interpreted using the following guidelines [small (*η*2*p* = 0.01–0.05), medium (*η*2*p* = 0.06–0.13), and large (*η*2*p* =  ≥ 0.14)^67^.

## Supplementary Information

Supplementary Information.

## Data Availability

The datasets used and/or analysed during the current study available from the corresponding author on reasonable request. Cell Signaling Technology Cat# 4376, RRID:AB_331772. National Institutes of Health, Bethesda, MD, RRID:SCR_002285; http://fiji.sc/Fiji).InfoStat, (RRID:SCR_014310). Thermo Fisher Scientific Cat# PA1-46,296, RRID:AB_2223196. Santa Cruz Biotechnology Cat# sc-17797, RRID:AB_628241 5. LI-COR Biosciences Cat# 926–32,222, RRID:AB_621844/48; National Institutes of Health, Bethesda, MD, RRID: SCR_002285.
