# Supplementary material for: Molecular neurobiological markers in the onset of sodium appetite
Source: Sci Rep. 2022 Aug 20;12:14224. doi: 10.1038/s41598-022-18220-w (PMC9392805; doi:10.1038/s41598-022-18220-w)
Supplement: Supplementary file 1 — Supplementary Information. [file 41598_2022_18220_MOESM1_ESM.docx]

**Title: MOLECULAR NEUROBIOLOGICAL MARKERS IN THE ONSET OF SODIUM APPETITE**

**Cintia Y. Porcari, María J. Cambiasso, André S. Mecawi, Ximena E. Caeiro, José Antunes-Rodrigues, Laura Vivas and Andrea Godino.**

**Supplementary Figure S1**


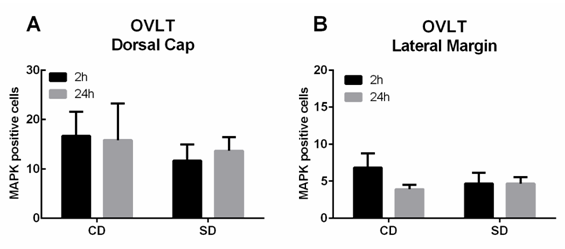


**Figure S1:** Average number of pERK1/2 positive neurons at two and 24 hours after sodium depletion in the OVLT dorsal cap (A) and lateral margin subdivision (B). Values are mean ± SE (n = 3-6/group). CD: Control group, SD: Sodium-depleted group.

**Supplementary Figure S2**


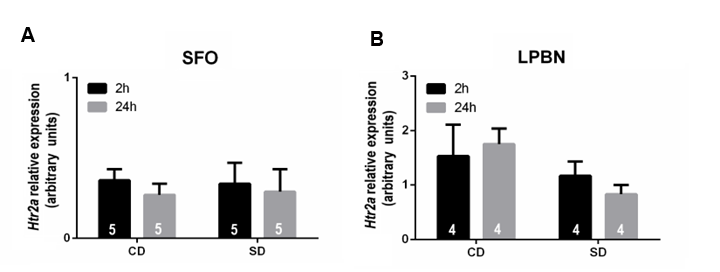


**Figure S2:** Relative serotonin 2a receptor (Htr2a) mRNA expression in SFO (A) and LPBN (B) at two and 24 hours after sodium depletion. Values are mean ± SE (number of cases indicated in the graphs) . SD: Sodium-depleted group. CD: Control group. LPBN: Lateral Parabrachial Nucleus. SFO: Subfornical Organ.

**Supplementary Figure S3**

| A- | B- |
| --- | --- |
| 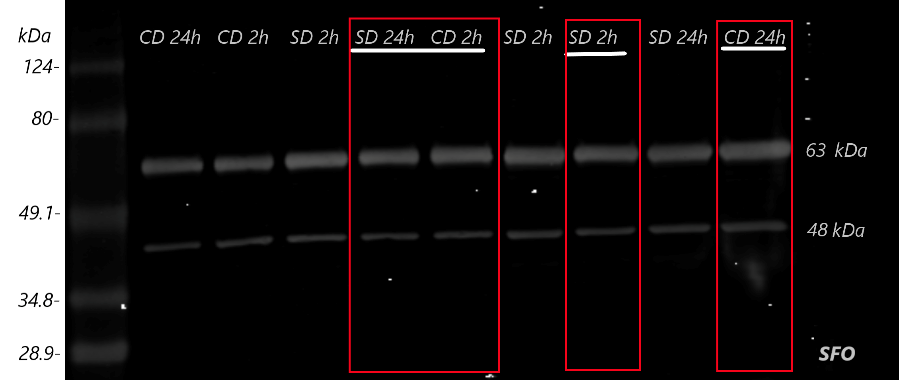 | 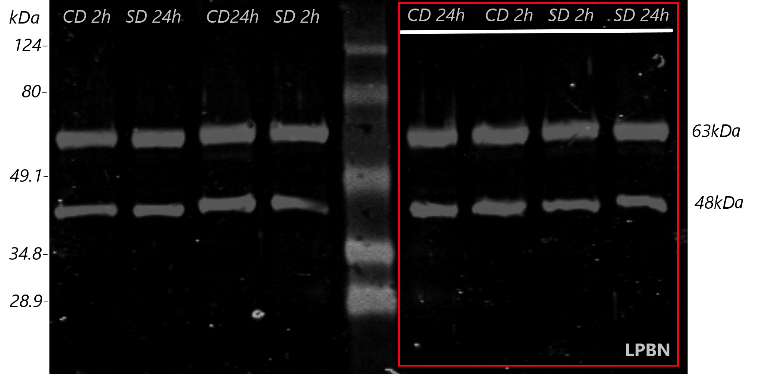 |
| C- |  |
| 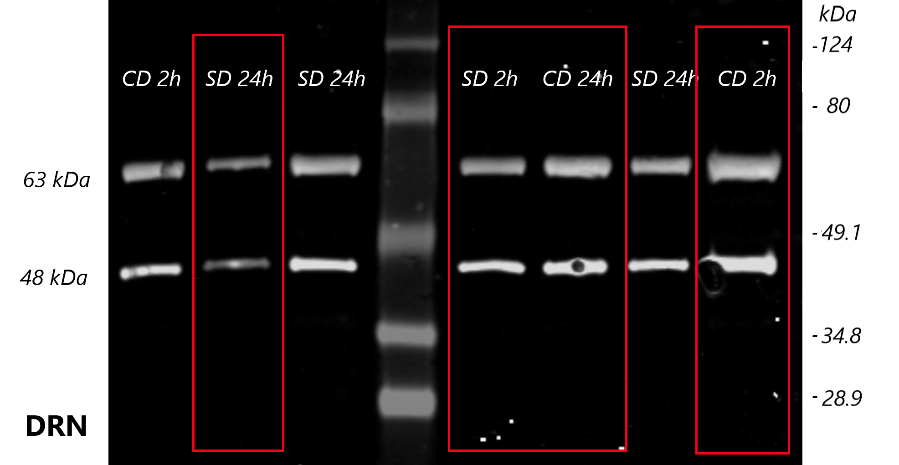 |  |
|  |  |

**Figure S3.** Uncropped blots of glycosylated (63kDa) and endogenous (48kDa) 5HT2c receptor in the subfornical organ (A), lateral parabrachial nucleus (B), and Dorsal Raphe Nucleus (C). Band intensity was quantified with NIH Image J software. The images were not modified. Underlined and red-boxes cases were taken for Fig. 5 of the manuscript. Images were switched to Black and White prior to analysis.

**Supplementary Figure S4**

| **A-** | **B-** |
| --- | --- |
| 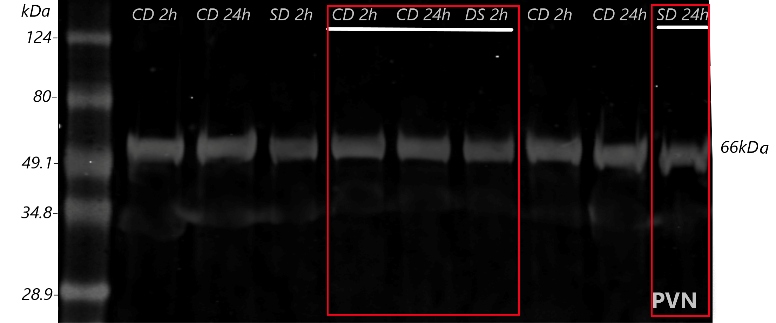 | 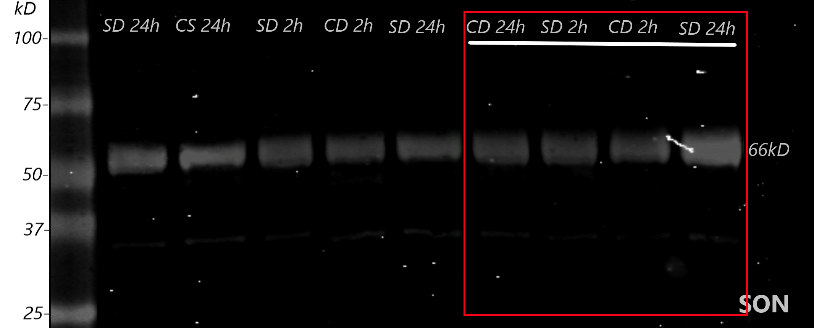 |
| **C-** | **D-** |
| 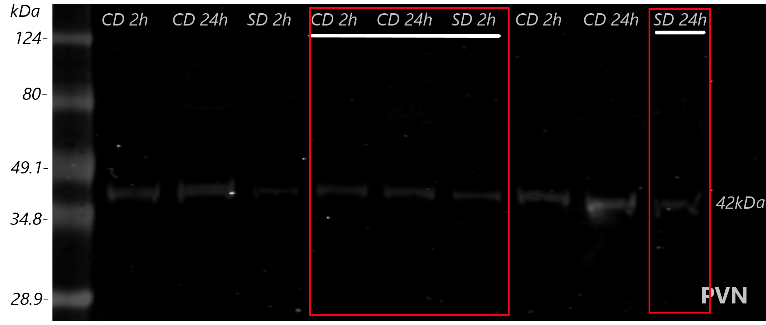 | 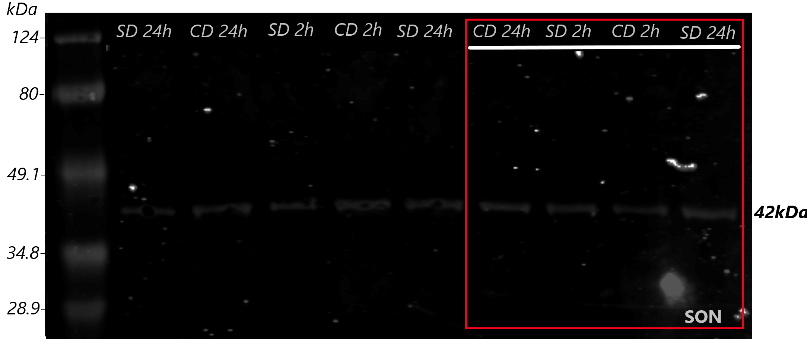 |

**Figure S4.** Uncropped blots of Oxytocin-Neurophysin I (A and B) and β-actin (C and D) along paraventricular (A and C) and supraoptic nucleus (B and D). Band intensity was quantified with NIH Image J software. The images were not modified. Underline and red-boxes cases were taken from Fig. 6 of the manuscript. Images were switched to Black and White prior to analysis.

**Supplementary Table S1**

Table 1: Plasma sodium, chloride and protein concentration and osmolality

| **Groups** | **[Na+]mEq/l** | **[Cl-]mEq/l** | **Plasma protein**  **gr/dl** | **Osmolality**  **mOsm/kg H_2_O** |  |  |
| --- | --- | --- | --- | --- | --- | --- |
| **SD 2 h** | 134,08 ± 3,13*  (n=4) | 73,24 ± 2,50*  (n=5) | 7,42 ± 0,13+  (n=4) | 280,50 ± 7,68*  (n=4) | | |
| **SD 24h** | 130.78 ± 0,39*  (n=4) | 73,50 ± 1,22*  (n=5) | 6,64 ± 0,15  (n=5) | 274,80 ± 4,64*  (n=5) | |  |
| **CD 2 h** | 139.88 ± 1,85  (n=4) | 88,70 ± 0,65  (n=4) | 6,16 ± 0,10  (n=4) | 296,00 ± 2,12  (n=4) | | |
| **CD 24h** | 142,85 ± 0,89  (n=4) | 90,80 ± 0,76  (n=4) | 6,43 ± 0,15  (n=4) | 300,75 ± 1,65  (n=4) | | |

*p<0.05 significant difference between SD vs.CD (sodium depletion factor)

+ p<0.05 significant difference between SD 2 h vs. other groups.

**Supplementary Table S2**

Table 2: Tests of Normal Distribution of analyzed variables

| **VARIABLES** | **Shapiro-Wilks** | | **Kolmogorov** | | **QQ-plot** |
| --- | --- | --- | --- | --- | --- |
|  | **W** | **p** | **D** | **p** | **r** |
| *Trpv1*-SFO | 0.92 | 0.4608 | 0.15 | 0.9309 | 0.956 |
| *Trpv1*-AV3V | 0.93 | 0.4383 | 0.11 | 0.9957 | 0.986 |
| *5htr2a*-SFO | 0.88 | 0.0555 | 0.25 | 0.9309 | 0.943 |
| *5htr2a*-LPBN | 0.92 | 0.3010 | 0.16 | 0.8185 | 0.950 |
| *Agtr1a*-SFO | 0.97 | 0.9192 | 0.26 | 0.1637 | 0.974 |
| *Agtr1a*-LPBN | 0.90 | 0.2154 | 0.25 | 0.3158 | 0.924 |
| *Agtr1a*-AV3V | 0.98 | 0.9720 | 0.15 | 0.9493 | 0.984 |
| *Agtr1a*-DRN | 0.96 | 0.8250 | 0.22 | 0.6252 | 0.976 |
| *5htr2c*-LPBN | 0.94 | 0.6615 | 0.15 | 0.8927 | 0.969 |
| *5htr2c*-SFO | 0.94 | 0.5970 | 0.14 | 0.8948 | 0.980 |
| *5htr2c*-AV3V | 0.95 | 0.7271 | 0.12 | 0.9947 | 0.988 |
| *5htr2c*-DRN | 0.98 | 0.9873 | 0.1 | 0.9968 | 0.988 |
| *Tph*2-DRN | 0.98 | 0.9742 | 0.13 | 0.9685 | 0.984 |
| *Sert*-DRN | 0.91 | 0.2425 | 0.22 | 0.4253 | 0.959 |
| 5Ht2c G/E-LPBN | 0.97 | 0.8849 | 0.12 | 0.8591 | 0.979 |
| 5Ht2c G/E-SFO | 0.95 | 0.6555 | 0.16 | 0.7740 | 0.979 |
| 5Ht2c G/E-DRN | 0.95 | 0.6776 | 0.12 | 0.9522 | 0.950 |
| *Oxtr*-AV3V | 0.91 | 0.3283 | 0.23 | 0.4702 | 0.912 |
| *Oxtr*-DRN | 0.90 | 0.3038 | 0.20 | 0.7120 | 0.967 |
| OXT-NP PVN | 0.95 | 0.6337 | 0.23 | 0.2813 | 0.952 |
| OXT-NP SON | 0.90 | 0.1171 | 0.17 | 0.6700 | 0.970 |
| ERK ½ -OVLT | 0.92 | 0.4095 | 0.18 | 0.8152 | 0.978 |
| ERK ½ -SFO | 0.98 | 0.9780 | 0.12 | 0.9973 | 0.987 |
| ERK ½- SON | 0.97 | 0.8949 | 0.20 | 0.7079 | 0.971 |
| ERK ½-PVN | 0.89 | 0.2348 | 0.29 | 0.2511 | 0.948 |
